# An ensemble method for prediction of phage-based therapy against bacterial infections

**DOI:** 10.3389/fmicb.2023.1148579

**Published:** 2023-03-23

**Authors:** Suchet Aggarwal, Anjali Dhall, Sumeet Patiyal, Shubham Choudhury, Akanksha Arora, Gajendra P. S. Raghava

**Affiliations:** ^1^Department of Computer Science and Engineering, Indraprastha Institute of Information Technology, New Delhi, India; ^2^Department of Computational Biology, Indraprastha Institute of Information Technology, New Delhi, India

**Keywords:** phage-host interaction, taxonomic levels, prediction, ensemble method, bacterial infection

## Abstract

Phage therapy is a viable alternative to antibiotics for treating microbial infections, particularly managing drug-resistant strains of bacteria. One of the major challenges in designing phage-based therapy is to identify the most appropriate potential phage candidate to treat bacterial infections. In this study, an attempt has been made to predict phage-host interactions with high accuracy to identify the potential bacteriophage that can be used for treating a bacterial infection. The developed models have been created using a training dataset containing 826 phage- host interactions, and have been evaluated on a validation dataset comprising 1,201 phage-host interactions. Firstly, alignment-based models have been developed using similarity between phage-phage (BLASTPhage), host–host (BLASTHost) and phage-CRISPR (CRISPRPred), where we achieved accuracy between 42.4–66.2% for BLASTPhage, 55–78.4% for BLASTHost, and 43.7–80.2% for CRISPRPred across five taxonomic levels. Secondly, alignment free models have been developed using machine learning techniques. Thirdly, hybrid models have been developed by integrating the alignment-free models and the similarity-scores where we achieved maximum performance of (60.6–93.5%). Finally, an ensemble model has been developed that combines the hybrid and alignment-based models. Our ensemble model achieved highest accuracy of 67.9, 80.6, 85.5, 90, and 93.5% at Genus, Family, Order, Class, and Phylum levels on validation dataset. In order to serve the scientific community, we have also developed a webserver named PhageTB and provided a standalone software package (https://webs.iiitd.edu.in/raghava/phagetb/) for the same.

## Introduction

Bacterial infections pose a major threat to public health across the globe. According to recent reports around, 1.27 million people died in 2019 of bacterial infections due to antimicrobial-resistance (Antimicrobial Resistance, 2022). In the last few decades, the heavy consumption and misuse of antimicrobial and antibacterial drugs have exacerbated the current crisis (Fair and Tor, 2014; Ventola, 2015). It has been observed in recent studies that several novel bacterial strains are emerging which are resistant to existing antibiotics (Magiorakos et al., 2012). Therefore, researchers are looking for alternative approaches to tackle this issue. One such approach is “phage therapy” where phages infect and lyse bacterial strains (Sulakvelidze et al., 2001; Lin et al., 2017; Furfaro et al., 2018; Gordillo Altamirano and Barr, 2019). One of the major challenges in designing phage therapy is to identify the most efficient bacteriophage that can lyse a target strain of bacteria (Roucourt and Lavigne, 2009; Yang et al., 2014). Currently, several techniques are available to measure the phage-host interactions such as RNA-sequencing, microfluidic-PCR, PhageFISH, and flow cytometry. In addition, spot test and agar overlay assay are used nowadays to match the phage-bacteria. Though these experimental techniques are highly accurate in identification of phage-bacteria interaction but they are costly and time consuming (Alvarez-Barrientos et al., 2000; Tadmor et al., 2011; Leskinen et al., 2016; Barrero-Canosa and Moraru, 2019; Grainha et al., 2020).

Thus, there is a need to develop computational methods that can predict the correct bacteriophage to treat a bacterial strain. In other words, there is a need to develop a method that can predict phage-host interaction (bacteriophage-bacteria) with high precision. In order to address this problem, a large number of methods have been developed in the past. Broadly, these methods can be classified into three categories - alignment-based, alignment-free and hybrid methods. The following are the brief description of major techniques developed in the past for predicting host-phage interaction. WIsH is an alignment-free tool that predicts prokaryotic hosts of phages using their genomic sequences (Galiez et al., 2017). VirHostMatcher-Net (Wang et al., 2020) is an hybrid method that combine several alignment-free and alignment-based features to construct a two-layered network model. SpacePHARER (Zhang et al., 2021) and VirSorter (Roux et al., 2015) use CRISPRs for predicting phage-host interaction in the prokaryotic genomes. PredPHI (Li et al., 2021) utilizes phage-host protein-based features for predicting phage-host interactions using deep convolutional networks. Despite several methods developed in the past decade for predicting phage-host interaction, their accuracy is far from satisfactory. Moreover, these existing methods do not provide user-friendly webserver facilities (Roux et al., 2015; Galiez et al., 2017; Wang et al., 2020; Li et al., 2021; Zhang et al., 2021). Hence, there is a challenge to develop methods that can predict phage-host interaction with high accuracy. In order to complement the existing methods, we have tried to develop an ensemble method for predicting phage-host interactions. Our proposed ensemble method combines alignment free (machine learning) and alignment-based (BLAST) techniques to predict phage-host interaction across all five taxonomic levels.

To maintain scientific standards and compare our approach with existing methods, we developed and evaluated our models on benchmark datasets used in a recent study (Wang et al., 2020). We have applied several machine learning techniques to develop the prediction models. One of the main objectives of this study is to facilitate researchers working in the field of phage therapy by identifying potential phage candidates that might be suitable to lyse drug-resistant bacterial strains and thus helping in narrowing down the search for suitable phages. Thus, we developed PhageTB (Webserver and Standalone Software) that contain three major modules; (i) host for a phage, (ii) phage-host interaction and (iii) phage for a host. The first module allows the users to predict the bacterial strain (i.e., host) from a phage genome sequence. The second module (Phage-host interaction) allows the user to predict whether a given phage and bacterial strain will interact or not. The third module, phage for a host, allows a user to predict the most appropriate phage that can lyse a given strain of bacteria.

## Materials and methods

### Dataset collection and pre-processing

**Figure 1:**
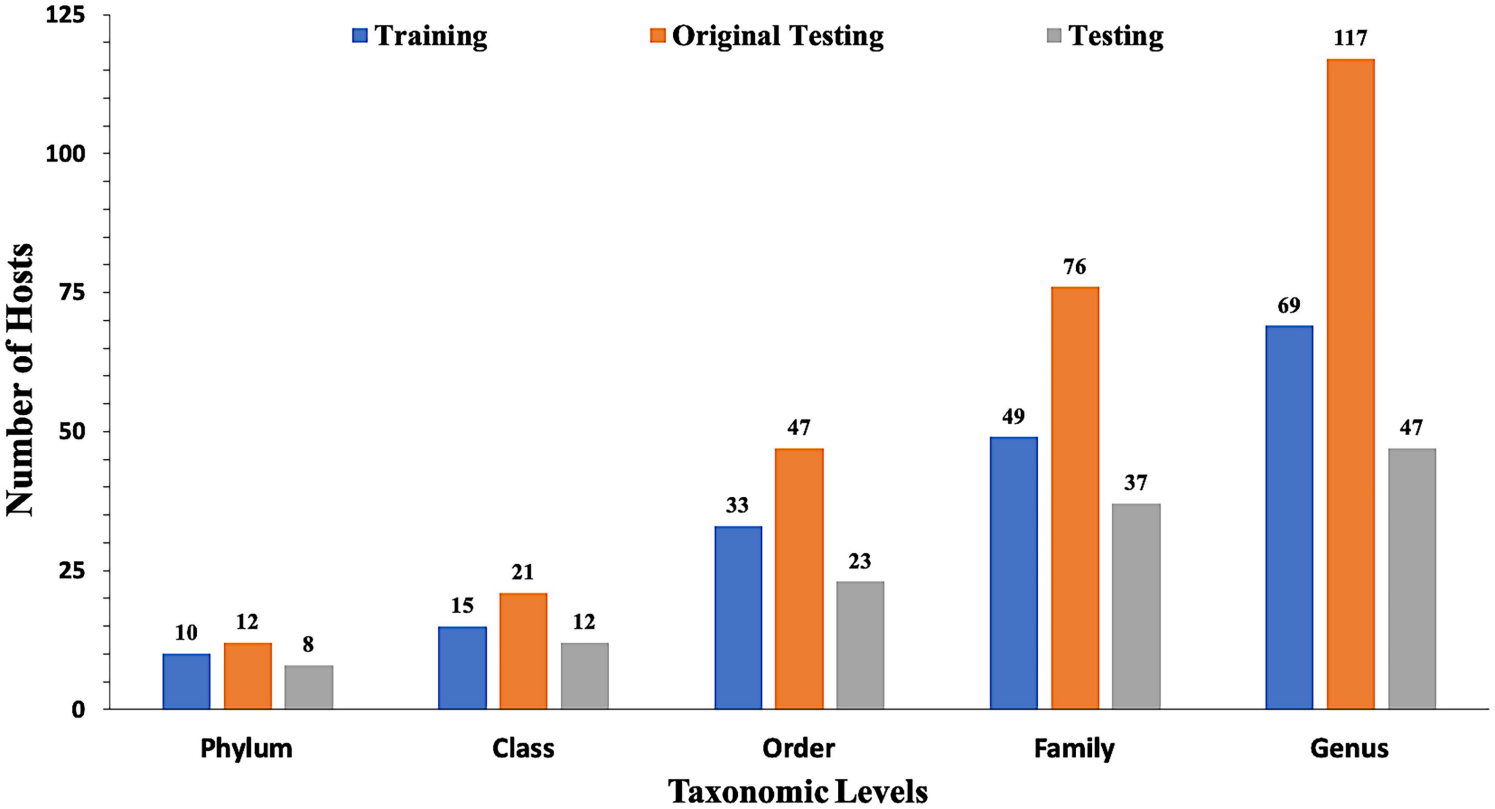
Distribution of data in training, testing, and original testing datasets at different taxonomic levels.

In the present study, datasets used for training and validation were obtained from a recent study VirHostMatcher-Net (Wang et al., 2020). The training dataset comprises 826 phages and their corresponding hosts (till the strain level), out of which 817 infect bacteria while nine infect archaea. The chosen dataset is such that each phage has a unique interaction with a bacterial strain. Aggregating the strain to a higher taxonomic level (till Genus, Phylum etc.) allows each phage to have multiple target hosts. Originally, we obtain around 1,462 phage entries and their corresponding hosts as the original testing dataset. However, the original testing dataset has one major issue that it contains phage-host pairs where some of the bacterial hosts belong to a genus that does not fall in the genera of the bacterial hosts of the training phage-host pairs. Evaluation of such phage-host interaction is not prudent as we do not have reference hosts representing such genera in the training phage-host pairs. Ideally, the test dataset should only contain the phage-host interactions, where the host information is available in our reference training data. Hence, we modified the original testing dataset and called it as the testing dataset, by removing the phage-host pairs whose hosts belong to a genus, not represented in the set of hosts from the training phage-host pairs. Out of the 1,462 phage-host pairs, there were 261 phage-host interactions which were not present in the training interactions. Hence, we removed 261 pairs and get 1,201 phage-host interactions in the testing dataset. Finally, our training dataset incorporates 826 phage-host interactions, and the testing dataset has 1,201 phage-host interactions. To make an unbiased comparison with the existing methods, we also evaluate our approach’s performance on the original testing dataset (See Supplementary materials). Figure 1 highlights the number of distinct Phylum, Classes, Orders, Families and Genera the bacterial hosts belong to, in at least one inter-action in the training, testing, and original testing datasets.

### Outline of the study

**Figure 2:**
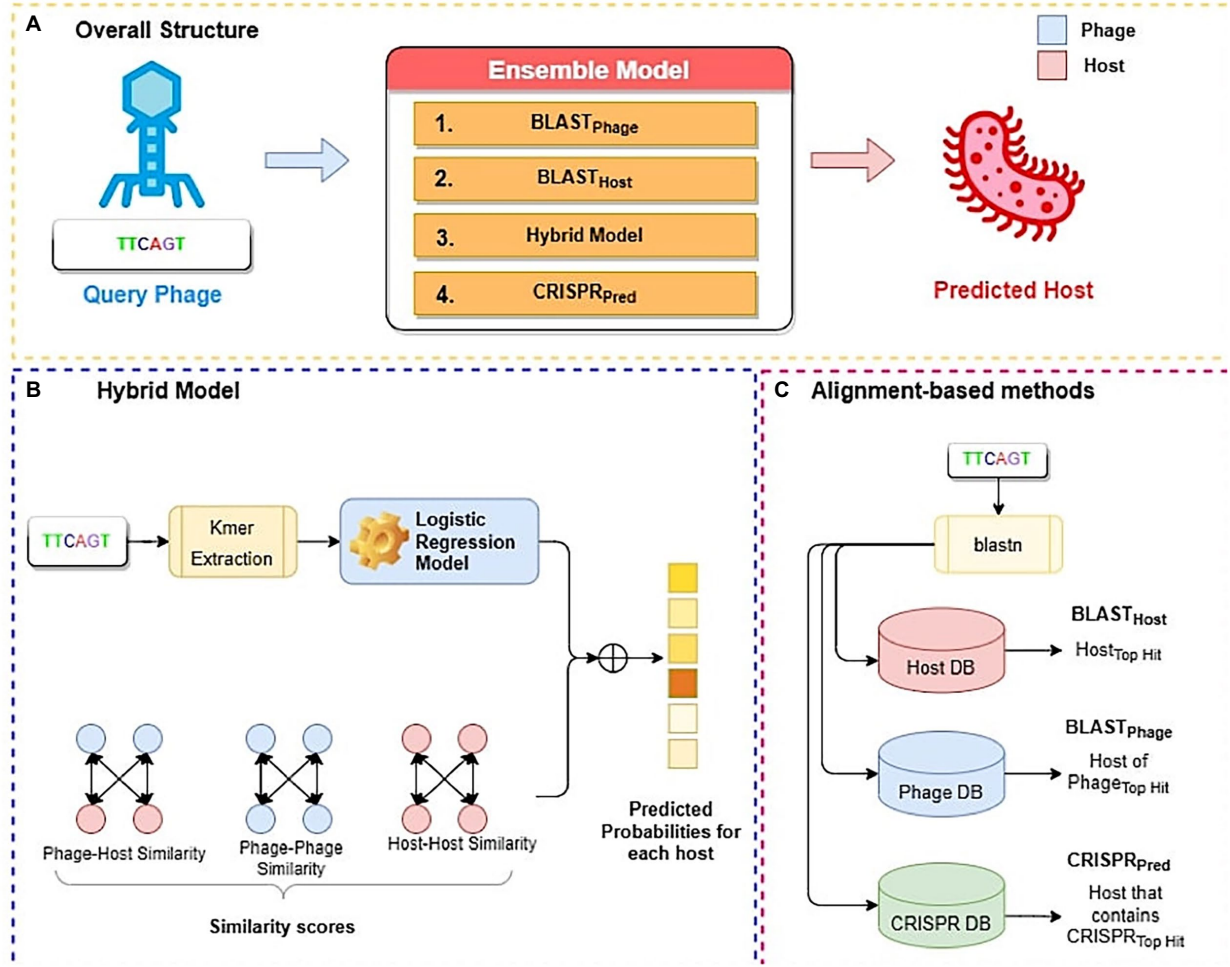
Overall structure of algorithms used in this study; **(A)** Ensemble method **(B)** Hybrid model **(C)** Alignment-based method.

In this study, we have developed three alignment-based methods using BLAST called BLASTHost, BLASTPhage and CRISPRPred (See Figure 2C). These alignment-based methods are based on top hits of BLAST search. Alignment based predictions are sometimes inadequate when we do not get any significant hit, in such cases alternative predictions can help. Therefore, we also create machine learning models that we used for predicting the hosts for phages. We also developed a hybrid method that combines a machine-learning based model with similarity scores (bit-scores from BLAST alignments) (See Figure 2B). Finally, an ensemble method has been developed that combines all alignment-based models with the hybrid method in a sequential method (Figure 2A). Predictions from the ensemble model are made in a staged sequential manner. First, predictions for all phages are made using BLASTPhage. We assign hosts corresponding to the top hit for the phages where the e-value of alignment is within a predetermined threshold. Next, for the remaining phages, we make the predictions using BLASTHost. We assign hosts to the phages where the e-value of alignment with the top hit is within a predetermined threshold. Third, for the phages whose hosts have not yet been assigned, we make predictions from the hybrid model and assign hosts for phages where the prediction scores from the model are above a threshold. Finally, for all remaining phages whose host could not be predicted, we assign hosts based on predictions from CRISPRPred.

### Alignment-based methods

Most of the alignment-based methods exploit sequence similarity between genomes of phages and their hosts. The most widely used method for searching for similar sequences is BLAST (McGinnis and Madden, 2004). We employed BLAST-based predictions at three levels, i.e., BLASTPhage, BLASTHost, and CRISPRPred. In the case of BLASTPhage, phage’s genome sequence is searched against a database of phages whose interacting host is already known. This database is referred to as the reference phage database in our study and it was created using training dataset comprising the information about the phages and their respective interacting hosts. Then, the phage sequences in the testing dataset are searched against the reference phage database using BLAST at different e-values. The host corresponding to the top BLAST hit of a phage is assigned as the predicted host for the query phage. In summary, the BLASTPhage model predicts the host based on similarity in the query and target phage. In the case of BLASTHost, the sequence of a phage is searched in database of 185 host sequences used in the training dataset. The top hit from this alignment task is assigned as the potential host. CRISPR systems play a vital role during the infection process of phages and infection prevention by the hosts. As a prevention strategy, prokaryotes place a fragment of the genome of an infecting phage as a spacer in the CRISPR array, which is a recognizable repeat region in the genome. Such a sequence indicates a recent infection and thus can be used as a potential signal for predicting hosts. CRISPR Recognition Tool (CRT) (Bland et al., 2007) is used to identify CRISPR locus in the bacterial genomes using a reference host database. We extracted CRISPR sequences using the CRT tool and created a reference CRISPR database. The test dataset genomes are aligned with the reference CRISPR database using BLAST, where the host corresponding to the top hit is predicted as the potential host. In the case of CRISPR alignment we have utilized the BLAST short-task parameter as used in a previous study (Biswas et al., 2013). We termed this approach of alignment as the CRISPRPred model.

### Generation of features

To develop machine-learning models for prediction, it is necessary to generate fixed-length feature representations for all phage sequences. The phage genome sequences are polymers of four nucleotides (A, T, G, C) and have a wide range of variations in length. One of the commonly used techniques to generate fixed-length feature representation for a sequence is to calculate the frequency of nucleotide sub-sequences or k-mers. For example, one can calculate the frequency of individual nucleotides in a sequence, and the sequence is thus represented by a vector of dimension four. In this case, the total number of k-mer is 4 (4^1^), where the subsequence or k-mer length is one. Similarly, the frequency of di-nucleotides (i.e., AA, AC, AG, AT, CA, CC) can be calculated, where the total number of k-mers will be 16 (4^2^), with the k-mer length being two. One of the limitations of these frequency-based features is that they are biased by the length of the sequence and the noise in the sequence. Thus, we used modified frequency words, subtracting the frequency of k-mers by chance in that sequence (Reinert et al., 2009). The following formulae were used to compute the modified frequency of k-mers, which is used.


(1) $$f_{m}=f_{o}-f_{c}$$

(2) $$f_{c}=P_{w}\times \left\{L-\left(k-1\right)\right\}$$

(3) $$P_{w}=\Pi_{i=1}^{k}p_{i}$$

Where *f_m_, f_0_*, and *f_c_* are modified, the original and chance frequency of a k-mer *w,* respectively. *P_w_* is the probability of k-mer w, *p_i_* is the probability of a nucleotide *i* in the k-mer *w*, *L* is the sequence length or the number of nucleotides in the sequence, and *k* is the length of k-mer *w.*

### Machine learning model

Several machine learning classifiers were implemented for predicting the hosts for bacteriophages and compared to develop the best-performing model. We have implemented various techniques including Random Forest (RF), Gaussian Naive Bayes (GNB), Logistic regression (LR), Support vector machine (SVM) with a linear kernel, eXtreme Gradient Boosting (XGBoost), Decision Tree (DT), K-Nearest Neighbor (KNN), and Multi-layer Perceptron (MLP). These classification techniques were implemented using the python-library scikit-learn (Pedregosa et al., 2012).

### Hybrid model

We utilize machine learning models at the third level for the remaining phages, i.e., those whose host could not be predicted using the BLASTPhage and BLASTHost method. We term this level of prediction as the hybrid model. Due to the coevolution of phages and their hosts, their genetic compositions are highly similar. Thus, a given phage significantly overlaps with its putative host at the genomic level. Therefore, similar hosts will likely be infected by the same phage, or similar phages will likely infect the same host. We have used the base machine-learning model prediction probabilities ${\mathrm{Pr}}_{b}=\left[{\mathrm{Pr}}^{i}\;\;for\;\;i=1,\ldots M\right]$ for all hosts, where M is the total number of hosts in the reference host database and ${\mathrm{Pr}}^{i}$ (prediction probability for the i^th^ bacteria) which varies between 0 to 1. In addition, we have added the similarity-scores (SIM) i.e., bit-scores from BLAST alignment tasks between phage-phage, phage-host, and host–host databases using a weighted sum to the prediction probabilities from the base machine-learning model. Further, we have used the ${\mathrm{Pr}}_{o}$ to calculate the final prediction probabilities for all bacterial hosts.


(4) $${\mathrm{Pr}}_{o}={\mathrm{Pr}}_{b}\left(1-\gamma \right)+\left(SIM_{PH}\left(v_{q},H\right)\left(1-\alpha \right)+SIM_{HH}\left(h_{s}\mathrm{,H}\right)\alpha \right)\gamma$$

Where, $SIM_{PP},SIM_{PH}\;and\;SIM_{HH}$ denote phage-phage, phage-host, and host–host similarity scores, where $SIM_{PH}\left(v,H\right)\;$ gives an M-Dimensional vector that gives the similarity scores of phage v with all hosts in the set $H=\left[h_{1},h_{2},\ldots h_{M}\right]$ of reference hosts. Similarly, $SIM_{HH}\left(h,H\right)$ also gives an M-Dimensional vector denoting the similarity of the host h with all other hosts in set H. $v_{q}\;$ corresponds to the input query phage, $h_{s}$ represents the host of most similar phage in the training dataset based on $SIM_{PP}\;$, and ${\mathrm{Pr}}_{b}\;$ is the prediction probabilities from the base model. Here, α and γ are the weighting parameters used in the given equation and are determined experimentally during cross-validation using grid search over the value range of 0 to 1 with step size of 0.1. The final predictions from the hybrid model were calculated using Equation 5.


(5) $$PredictedHost\left(v\right)=argmax_{h}\;\;{\mathrm{Pr}}_{o}\left(h\right)$$

### Ensemble model

In order to improve the prediction accuracy, without compromising the coverage we have used an ensembled approach, generating predictions using combinations of different models. Here, we integrate alignment-based, alignment-free models. At first, we calculate predictions from BLASTPhage and assign host against phages where the e-value of alignment is within a threshold. Similarly, this process was repeated for remining phages using BLASTHost. Next, we compute predictions from the hybrid model for phages where final prediction score is above a threshold (See Equation 5). Finally, for the remaining phages, predictions are made using CRISPRPred.

### Evaluation parameters

We evaluate the performance of our approach on the original testing dataset, which comprises 1,462 phage samples. Moreover, we have also evaluated the performance of the generated models on the modified testing dataset containing 1,201 phage samples. We also compare our approach with past studies in terms of prediction accuracy for correctly predicting hosts binned by taxonomic levels from Genus to Phylum. The prediction accuracy is defined as the fraction of phages whose hosts were identified correctly out of the total phages at a given taxonomic level.


(6) $$Accuracy=\frac{NumberofCorrectPredictions}{Totalnumberoftestsamples}\times 100$$

(7) $$\begin{array}{l} Probabilityofcorrectprediction\\ =\frac{NumberofCorrectPredictions}{Totalnumberofpredictions}\times 100 \end{array}$$

### Webserver architecture

A web server named as ‘PhageTB’^1^ is developed to predict the bacterial hosts, host-phage interactions, and lytic phage for a bacterium. The front end of the web server was developed by using HTML5, JAVA, CSS3 and PHP scripts. It is based on responsive templates which adjust based on the size of the device. It is compatible with almost all modern devices such as mobile, tablet, iMac, and desktop.

## Results

### Predictions from BLASTPhage, BLASTHost and CRISPRPred

**Figure 3:**
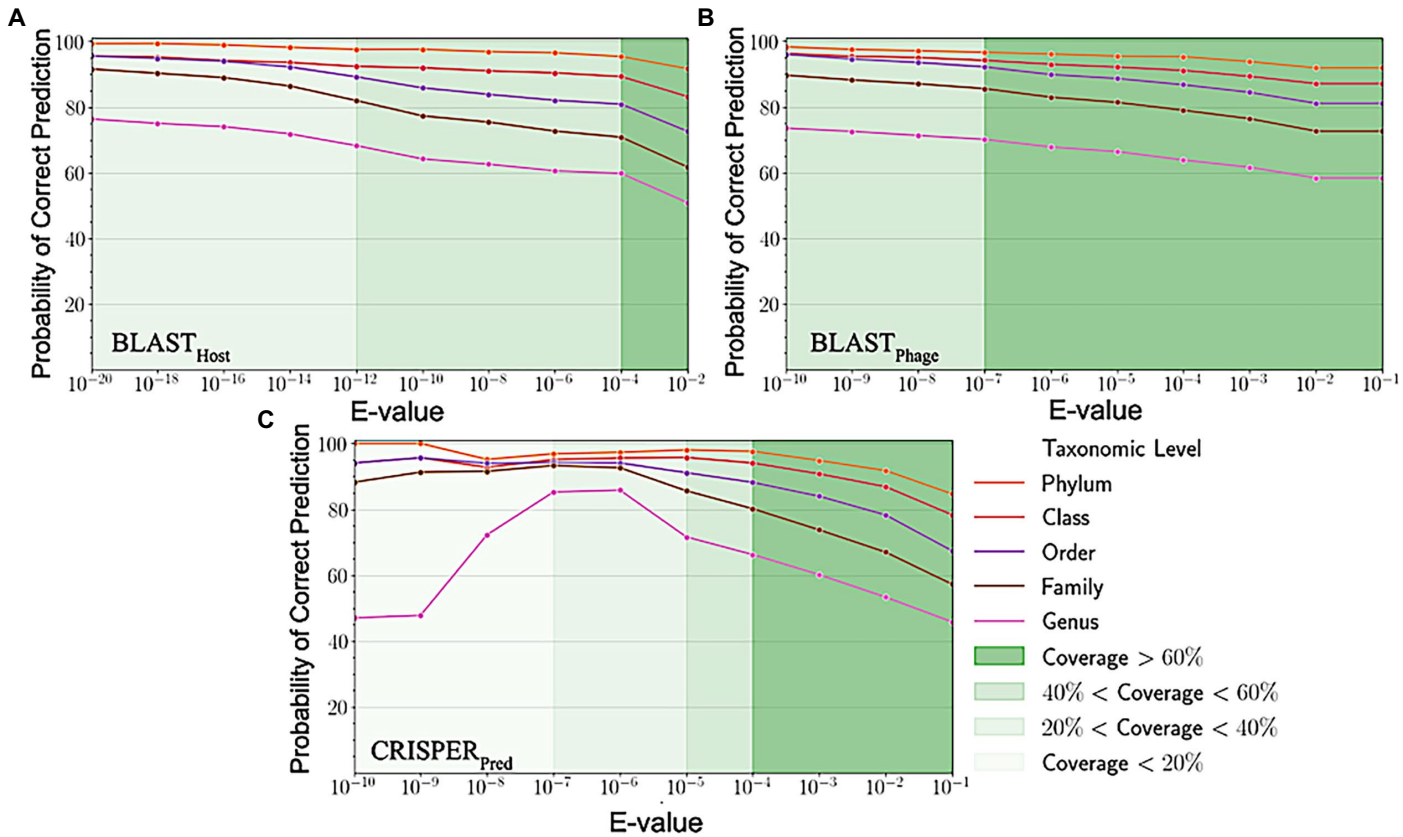
Variation in probability of correct prediction **(A)** BLAST_Phage_ **(B)** BLAST_Host_ and **(C)** CRISPR_Pred_ at different e-values.

Sequence alignment of phage and host genomes is the primary method for assigning hosts to phages from a set of known hosts. For this purpose, we employed BLAST technique, where first we vary the degree of alignment by changing the threshold on the e-value. For the query phages where we get a sequence match, we observe the prediction accuracies improved when the e-value threshold is reduced, but the overall recall decreases. However, we could not predict hosts using this method for all phages, as shown in Figure 3, the coverage decreases as we decrease the e-value threshold and the sequence match becomes more specific. When aligning the phage genomes in the original testing dataset with the reference phage genome database, and assigning the host based on the top hit, we attained the prediction accuracies of 45.2, 56.2, 62.8, 67.5, and 71.2% at Genus, Family, Order, Class and Phylum levels, respectively (Supplementary Table S1).

Similarly, on aligning the phage genomes with the reference host genome database and assigning the top hit as the predicted host, we obtained accuracies of 34.8, 42.3, 49.7, 57.0, and 62.8% at Genus, Family, Order, Class, and Phylum levels, at e-value 1.00E-02 (Supplementary Table S1). As reported in Table 1, we obtained accuracies of 42.4, 50.5, 57.2, 61.4, and 66.2% across the five taxonomic level using BLASTHost method at e-value 1.00E-02. Similarly, BLASTPhage attained accuracies of 55.0, 66.4, 71.4, 74.9, and 78.4% at Genus, Family, Order, Class, and Phylum levels, respectively on the test dataset. Further predictions were made by aligning phage genomes with CRISPR sequences extracted from host genomes. The predictions from CRISPRPred were very accurate for smaller e-value thresholds indicating precise predictions up to the Genus level, but at the same time the coverage (fraction of phages for which predictions could be made) was relatively small. This implied that although highly accurate predictions can be made using CRISPR signals but such predictions are not possible for all phages. We observe that in the case of the original test dataset (See Supplementary Table S1) and modified test datasets, the prediction accuracies were improved at the level of class and phylum in comparison with BLASTHost and BLASTPhage methods (refer to Table 1).

**Table 1:** Prediction of five taxonomic levels of bacterial host using alignment-based models on validation dataset.

E-value	Method	Cov (%)	Taxonomic Level									
			Genus		Family		Order		Class		Phylum	
			PCP(%)	Acc(%)	PCP(%)	Acc(%)	PCP(%)	Acc(%)	PCP(%)	Acc (%)	PCP (%)	Acc (%)
1.00E-06	*BLAST_Host_*	50.45	67.00	33.81	79.21	39.97	87.29	44.05	90.43	45.63	96.53	48.71
	*BLAST_Phage_*	70.77	74.12	52.46	88.24	62.45	93.41	66.11	95.53	67.61	98.24	69.53
	*CRISPR_Pred_*	21.57	87.64	18.90	92.66	19.98	93.82	20.23	96.14	20.73	97.30	20.98
1.00E-05	*BLAST_Host_*	53.46	66.98	35.80	78.82	42.13	87.07	46.54	90.34	48.29	96.26	51.46
	*BLAST_Phage_*	72.11	72.98	52.62	87.07	62.78	92.49	66.69	94.69	68.28	97.58	70.36
	*CRISPR_Pred_*	38.30	70.22	26.89	85.00	32.56	91.09	34.89	96.30	36.89	98.04	37.55
1.00E-04	*BLAST_Host_*	58.28	67.14	39.13	78.29	45.63	86.86	50.62	90.00	52.46	96.00	55.95
	*BLAST_Phage_*	75.52	70.67	53.37	85.12	64.28	90.74	68.53	93.72	70.77	97.35	73.52
	*CRISPR_Pred_*	51.87	66.45	34.47	81.22	42.13	90.37	46.88	95.83	49.71	97.59	50.62
1.00E-03	*BLAST_Host_*	64.45	63.31	40.80	74.81	48.21	84.75	54.62	89.41	57.62	95.22	61.37
	*BLAST_Phage_*	77.10	69.76	53.79	84.23	64.95	89.96	69.36	93.30	71.94	97.19	74.94
	*CRISPR_Pred_*	62.53	60.59	37.89	74.43	46.54	85.75	53.62	92.01	57.54	94.67	59.20
1.00E-02	*BLAST_Host_*	71.36	59.51	42.46	70.83	50.54	80.16	57.20	86.11	61.45	92.88	66.28
	*BLAST_Phage_*	81.77	67.31	55.04	81.57	66.69	87.37	71.44	91.65	74.94	95.93	78.43
	*CRISPR_Pred_*	73.77	54.40	40.13	68.62	50.62	80.59	59.45	88.94	65.61	92.44	68.19
1.00E-01	*BLAST_Host_*	71.44	59.44	42.46	70.75	50.54	80.07	57.20	86.01	61.45	92.77	66.28
	*BLAST_Phage_*	81.77	67.31	55.04	81.57	66.69	87.37	71.44	91.65	74.94	95.93	78.43
	*CRISPR_Pred_*	90.67	46.83	42.46	58.59	53.12	69.15	62.70	79.89	72.44	85.40	77.44

Cov(%): Coverage in percentage; Acc(%): Accuracy in percentage; PCP(%): Probability of Correct Prediction

### Performance of machine learning models

In order to develop various machine learning models, i.e., Decision Tree (DT), Random Forest (RF), Gaussian Naive Bayes (GNB), XGBoost, Logistic Regression (LR), Multi-layer perceptron (MLP), and Support Vector Machine (SVM), we extracted the features $f_{m}\;$ using Equation 1 with k = 6, from the phage genomes and using these features, we predicted the bacterial hosts for phages in the testing dataset. As represented in Table 2, on modified test dataset, LR-based models performed best among all other classifiers. In order to improve the performance further, we integrated the prediction score of the best model, i.e., LR with similarity scores, i.e., BLAST bit scores and observed that there is a significant improvement in the predictive accuracies. The parameters for the hybrid model were found by varying the weighting parameters α and γ in Equation 4. We achieved the best performance at *α* = 0.9 and *γ* = 0.6. On original test dataset, the prediction accuracies of the hybrid model (LR + similarly score) are 49.7, 64.7, 75.3, 84.8, and 90.6% across the five taxonomic levels, respectively, (Supplementary Table S2). On the other side, the hybrid model evaluated on the modified test dataset, outperformed all other classifiers with an improved accuracies of 60.6, 75.8, 82.0, 89.7, 93.5% at Genus, Family, Order, Class, and Phylum levels, respectively, (See Table 2).

**Table 2:** Prediction of five taxonomic levels of bacterial host using machine learning and hybrid models on modified test dataset.

Machine learning methods	Taxonomic levels (Accuracy %)				
	Genus	Family	Order	Class	Phylum
Decision Tree (DT)	22.30	30.50	34.70	45.70	55.90
Gaussian Naive Bayes (GNB)	24.30	32.00	35.20	42.90	45.60
XGBoost (XGB)	37.20	44.20	48.20	53.20	57.80
Random Forest Classifier (RF)	38.80	46.10	51.00	55.70	59.20
Linear SVM (SVM)	51.80	62.10	65.60	71.00	73.50
K-Nearest Neighbor (KNN)	49.20	61.60	67.60	73.20	78.50
Multi-layer perceptron (MLP)	49.20	63.50	69.10	76.10	80.00
Logistic Regression (LR)	54.80	68.10	72.50	77.70	80.80
Hybrid Model (Similarly-scores + LR)	60.60	75.80	82.00	89.70	93.50

### Performance of ensemble models

In order to improve the performance of the models mentioned above, we have used the ensemble approach, where we have generated predictions from combinations of the different models. Here, we have tried combinations of BLAST_Phage_, BLAST_Host_, CRISPR_Pred_ and the Hybrid Model and validated the accuracies at different taxonomic levels on both the testing datasets. We observe improvements across all the taxonomic levels as we progressively add the different prediction methods to the overall framework. Host prediction accuracy was markedly higher than individual components. For higher-order taxonomic levels (Class and Phylum) combination of BLAST and the hybrid model-based predictions also got comparable results. However, for lower and more specific levels, the best- performing approach was the one that combines all prediction methods. Our proposed ensembled model (BLASTPhage + BLASTHost + CRISPRPred + Hybrid Model) outperforms the existing approaches across all taxonomic levels, correctly predicting 61.6, 74.4, 80.5, 85.7, and 91.2%, respectively, for original test dataset (Supplementary Table S3) and 67.9, 80.6, 85.5, 90.0, and 93.5% for test dataset at Genus, Family, Order, Class and Phylum levels. The e-value thresholds for BLASTPhage (1.00E-10), BLASTHost (1.00E-20), CRISPRPred (1.00E-2), and the prediction probability threshold for the hybrid models is 0.6 (Table 3).

**Table 3:** Prediction of five taxonomic levels of bacterial host using ensembled models.

Method	Taxonomic Level (Accuracy %)				
	Genus	Family	Order	Class	Phylum
BLAST_Phage_ + BLAST_Host_	59.40	70.10	73.90	75.50	78.60
BLAST_Host_ + Hybrid Model	65.00	78.10	83.10	89.90	93.90
BLAST_Phage_ + Hybrid Model	62.60	75.80	82.40	89.80	93.60
CRISPR_Pred_ + Hybrid Model	61.10	74.10	80.50	86.40	90.10
BLAST_Phage_ + BLAST_Host_ + Hybrid Model	65.70	78.60	84.30	90.70	94.00
BLAST_Host_ + CRISPR_Pred_ + Hybrid Model	65.50	76.60	81.70	86.80	90.90
BLAST_Phage_ + CRISPR_Pred_ + Hybrid Model	66.70	79.20	84.60	89.80	93.50
BLAST_Phage_ + BLAST_Host_ + CRISPR_Pred_ + Hybrid Model	67.90	80.60	85.50	90.00	93.50

### Contributions to the scientific community

To serve the scientific community, we integrate our best-performing models in a webserver called “PhageTB.” This tool incorporates three major modules (i) Hosts for bacteriophages (ii) Interaction of phage-host pair and (iii) Lytic phage for a bacterial host. The first module “Hosts for bacteriophages” allows users to choose four predictive methods, i.e., BLASTPhage, BLASTHost, CRISPRPred, and Hybrid Model. Users need to provide the query genome sequence and the tool predict the bacterial hosts using the reference host database. The second module “Interaction of phage-host pair” predicts whether a pair of phage and bacteria are likely to interact based on their genome sequences. Users need to provide genome sequences of phage and bacteria in the FASTA format. Our tool predicts the interactions between the query sequences, by first, predicting the host of the query phage using the first module and then using sequence alignment between the predicted and query hosts to determine whether the query pair interact or not. The third module “Lytic phage for a bacterial host” predicts bacteriophages corresponding to query bacterial sequences. The input genome sequence is searched against the reference database of phage-host interactions, where first we align the query sequence with genome sequences of bacteria that are known hosts for some bacteriophages. The top hit bacteria from the reference database are the most similar bacteria to the query, and thus the query is likely to be infected by the phage associated with the top hit. The webserver “PhageTB” was implemented using HTML, CSS, and PHP and has multi-device compatibility, and provides an easy-to-use and user-friendly interface. The open-source web server is available at https://webs.iiitd.edu.in/raghava/phagetb. The command line standalone can be found on GitHub at https://github.com/raghavagps/phagetb.

### Case study: Prediction of lytic phages

Predicting lytic phages that can be used (solely or with other agents) for treatment of multi-drug resistance bacterial infections is a major problem of concern for the scientific community (Lin et al., 2017; Kortright et al., 2019). In this case study we identify suitable phage-based treatments for drug-resistant bacterial infections using our webserver PhageTB to predict the lytic phages corresponding to the six ESKAPE *Enterococcus faecium*, *Staphylococcus aureus*, *Klebsiella pneumoniae*, *Acinetobacter baumannii*, *Pseudomonas aeruginosa*, and *Enterobacter* species (Santajit and Indrawattana, 2016; Mulani et al., 2019) bacteria. ESKAPE comprises six well-known highly virulent antibiotic-resistant bacterial pathogens. Here we have downloaded the genome assemblies of each of the six bacteria from NCBI^2^ and predict the specific phage. We utilize the default parameters of the third module, “Lytic phage for a bacterial host” of PhageTB, to predict the phages that are likely to infect a bacterium. Table 4 and Supplementary Table S4 represents the predicted phages with GenBank ID for five out of six ESKAPE bacteria. We could not predict any lytic phage against Pseudomonas aeruginosa bacteria, which could be attributed to the use of strict thresholds for the individual models. We have evaluated the predictions of our tool with existing studies and clinical trials (El Haddad et al., 2019; Mulani et al., 2019). These findings can be extended to other drug-resistant bacterial strains and thus utilized to expedite the process of finding suitable phages for the treatment of drug-resistant bacterial infections where the lytic phages are not known beforehand.

**Table 4:** Lytic phage prediction by phageTB on ESKAPE bacteria.

Bacteria	Predicted phage (GenBank ID)	Evidence (Ref)
*Enterococcus faecium*	AB746912	Lee et al. (2019)
*Staphylococcus aureus*	DQ289556	Fish et al. (2016)
*Klebsiella pneumoniae*	CP000711	Manohar et al. (2018)
*Acinetobacter baumannii*	AB746912	Badawy et al. (2020)
*Pseudomonas aeruginosa*	No-Prediction	-
*Enterobacter*	CP000711	Manohar et al., 2018

### Comparison with other methods

**Figure 4:**
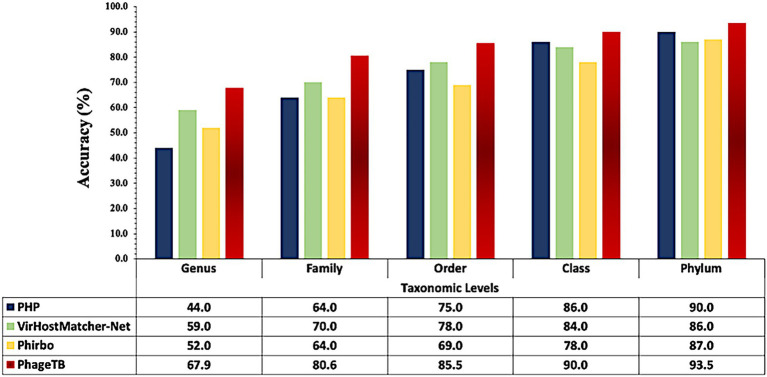
Comparison of performance of our method with existing tools at different taxonomic levels.

Comparing this newly developed method with the existing tools is crucial to understand the merits and demerits. There are several methods available such as VirHostMatcher-Net, PHP, Phirbo, and PredPHI as shown in Table 5. Therefore, we compare the performance of our method with three existing tools PHP (Lu et al., 2021), VirHostMatcher-Net (Wang et al., 2020), and Phirbo (Zielezinski et al., 2021), due to the prediction at all five taxonomic levels is available in only these tools. As shown in Figure 4, PhageTB outperform previous studies at each taxonomic level, with an accuracy of 67.90, 80.60, 85.5, 90.0, and 93.5% at Genus, Family, Order, Class, and Phylum levels. The prediction accuracies of other tools are provided in Figure 4.

**Table 5:** Comparison of PhageTB with the existing phage-host interaction prediction methods.

Tool	PhageTB	PHP	VirHostMatcher-Net	Phirbo	PredPHI
Webserver	Yes	No	No	No	No
Standalone	Yes	Yes	Yes	Yes	Yes
Genus	Yes	Yes	Yes	Yes	No
Family	Yes	Yes	Yes	Yes	No
Order	Yes	Yes	Yes	Yes	No
Class	Yes	Yes	Yes	Yes	No
Phylum	Yes	Yes	Yes	Yes	No
Phage2Host	Yes	Yes	Yes	Yes	Yes
Host2Phage	Yes	No	Yes	No	No
Phage2Phage	Yes	No	Yes	No	No

## Discussion and conclusion

Phage therapy is a leading alternative to antibiotics for the treatment of bacterial infections as most pathogenic strains are now showing resistance to numerous known antibiotics (Topka-Bielecka et al., 2021). The development of phage therapy requires the identification and isolation of a large number of bacteriophages. Phages are generally specific to bacterial species as well as their strains which is an advantage of this therapy as it will only kill the pathogenic bacteria, leaving out the natural bacteria required for the human body. The highly specific nature of bacteriophages necessitates the collection and characterization of their known and potential hosts and the interactions between them (Gorski et al., 2018, 2020). There have been several studies in the past that have tried to identify and predict the hosts of phages and their interactions like WIsH, VirHostMatcher-Net, SpacePHARER, VirSorter, and PredPHI (Roux et al., 2015; Galiez et al., 2017; Wang et al., 2020; Li et al., 2021; Zhang et al., 2021). Despite this, the presently available methods cannot accurately predict the taxonomic classes of the phage and hosts. To bridge this gap and achieve better performance in predicting the phage-host interactions, we developed a method called PhageTB that uses both alignment-based and alignment-free features to predict the hosts from query genomic sequences of bacteriophages.

PhageTB is a hierarchical prediction method that stacks four predictive methods to predict the phage-host interactions across five levels–Genus, Family, Order, Class, and Phylum. These methods include BLASTPhage, BLASTHost, the Hybrid model, and CRISPRPred. BLASTPhage, BLASTHost, employ BLAST alignment-based predictions for query sequences against reference hosts and phages, respectively, and the CRISPRPred approach uses CRISPR based alignment to predict the same. In cases where there is a shortage of phage or bacterial sequence data, traditional alignment-based methods may be unreliable in predicting rare phage-host interactions. However, machine learning models can be used to address this limitation. The Hybrid Model predicts the host based on the machine learning classifier and similarity scores. These four methods combined can accurately predict the host-phage interactions and outperform the previously developed methods to predict phage-host interactions namely PHP, VirHostMatcher-Net, and Phirbo when tested on the dataset containing 1,462 phage-host interactions (Wang et al., 2020; Lu et al., 2021; Zielezinski et al., 2021). We obtained accuracies of 67.9, 80.6, 85.5, 90.0, and 93.5% for Genus, Family, Order, Class, and Phylum, respectively, using the ensemble model which is better than the abovementioned methods. Additionally, it must be noted that the proposed tool has limitations regarding its ability to predict the evolution of bacterial resistance to phages, as it assumes that any phage used in the prediction can infect any bacteria without taking into consideration any developed resistance and does not explicitly identify phage resistance. The approaches combined in PhageTB provide accurate predictions for phage-host interactions making it a valuable tool for the scientific community working in this field worldwide to identify phages that might be suitable to combat the crisis of antibiotic resistance. With the increasing availability of metagenome samples, new methods for identifying phages and determining their hosts are required. We believe that PhageTB will prove to be an effective tool in finding specific hosts for the phages which can be potentially helpful in the development of phage therapy by facilitating as a useful filter to narrow down target phages and hosts, ecology research, viral metagenomics analysis, and human gut microbiocenosis research among others. PhageTB is an easy-to-use method of assigning hosts to bacteriophages, studying their interactions, and narrowing down the search space for candidate phages that can successfully lyse the query bacteria and thus be utilized in phage therapy for treating bacterial infections caused by it. Our tool is freely accessible at https://webs.iiitd.edu.in/raghava/phagetb/, and the Python standalone package is available at GitHub https://github.com/raghavagps/phagetb.

### Limitation of the study

In the current study, we have developed an in-silico tool for the prediction of phage-host interactions using ensemble learning approach. Due to the limitation in the available datasets we have not considered phage-host receptors and prophages for developing the prediction models. Moreover, we were not able to discriminate interacting and bacterial resistant strains. In future, we will update this tool by incorporating new features and experimentally validated data, in order to generate a highly accurate and reliable method for designing phage-based therapy.

## Data availability statement

The original contributions presented in the study are included in the article/Supplementary material, further inquiries can be directed to the corresponding author.

## Author contributions

SA, and GR collected and processed the datasets and implemented the algorithms. SA, AD, and SP created the back end of the web server and front-end user interface. SA developed prediction models. SA, SP, SC, and AD analyzed the results. SA, AD, SC, AA, SP, and GR penned the manuscript. GR conceived and coordinated the project and provided overall supervision to the project. All authors have read and approved the final manuscript.

## Funding

This research was funded by Department of Biotechnology (DBT grant BT/PR40158/BTIS/137/24/2021), Government of India, India.

## Conflict of interest

The authors declare that the research was conducted in the absence of any commercial or financial relationships that could be construed as a potential conflict of interest.

## Publisher’s note

All claims expressed in this article are solely those of the authors and do not necessarily represent those of their affiliated organizations, or those of the publisher, the editors and the reviewers. Any product that may be evaluated in this article, or claim that may be made by its manufacturer, is not guaranteed or endorsed by the publisher.

## Footnotes

^1^ https://webs.iiitd.edu.in/raghava/phagetb/

^2^ https://www.ncbi.nlm.nih.gov/assembly/
